# High rate of extended-spectrum beta-lactamase-producing gram-negative infections and associated mortality in Ethiopia: a systematic review and meta-analysis

**DOI:** 10.1186/s13756-020-00782-x

**Published:** 2020-08-08

**Authors:** Tafese B. Tufa, Andre Fuchs, Takele B. Tufa, Loraine Stötter, Achim J. Kaasch, Torsten Feld, Dieter Häussinger, Colin R. Mackenzie

**Affiliations:** 1 Hirsch Institute of Tropical Medicine, P.O. Box 04, Asella, Ethiopia; 2 College of Health Sciences, Arsi University, P.O. Box 04, Asella, Ethiopia; 3 Department of Gastroenterology, Hepatology and Infectious Diseases, Duesseldorf University Hospital Center, Moorenstr. 5, 40225 Duesseldorf, Germany; 4 College of Veterinary Medicine and Agriculture, Addis Ababa University, Bishoftu, Ethiopia; 5 Institute of Medical Microbiology and Hospital Hygiene, Düsseldorf University Hospital Centre, Universitätsstr. 1, 40225 Düsseldorf, Germany; 6 Institute of Medical Microbiology and Hospital Hygiene, Magdeburg University Hospital, Otto-von-Guericke-University, Leipziger Str. 44, 39120 Magdeburg, Germany

**Keywords:** ESBL, Gram-negative bacteria, Multidrug-resistance, Antimicrobial resistance

## Abstract

**Background:**

Extended-spectrum beta-lactamase (ESBL)-producing Gram-negative bacteria have become a serious threat to global health. Their rapid spread is associated with high mortality due to ineffective antibiotic treatment. To date a regular surveillance of multidrug-resistant (MDR) pathogens in Ethiopia is not established. For this report, published data regarding ESBL-producing bacteria in different health facilities of Ethiopia were reviewed.

**Methods:**

This study collates data from published information on the rates and clinical implications of infection with ESBL-producing Gram-negative bacteria in Ethiopia. A systematic literature search was conducted using PubMed, PubMed Central, Medline, Science Direct and Google scholar from October 2018 to March 2019. Eligible studies were identified by applying quality criteria. The pooled proportion of ESBL-producing Gram-negative bacteria was estimated based on a random effect model. The publication bias and the variation in proportion estimates attributed to heterogeneity were assessed.

**Results:**

Fourteen studies with relevant data were included in the review. In total, 1649 Gram-negative bacteria isolated from 5191 clinical samples were included. The pooled proportion estimate of ESBL-producing Gram-negative bacteria was 50% (95% CI: 47.7–52.5%. Data showed a high level of heterogeneity (I^2^ = 95%, *P* <  0.01). ESBL rates varied by species; 65.7% (263/400) in *Klebsiella* spp., 48.4% (90/186) in *Salmonella* spp., and 47.0% (383/815) in *E. coli*. ESBL-encoding genes were reported in 81 isolates: 67 isolates harbored the CTX-M-1 group and 14 isolates TEM. The mortality associated with infections by bacteria resistant to third generation cephalosporins has rarely been investigated. However, two studies reported a mortality of 33.3% (1/3) and 100% (11/11).

**Conclusions:**

In this meta-analysis, the pooled prevalence of ESBL-producing pathogens is alarmingly high. Data on mortality rates is scarce. This highlights the need for establishing and upgrading clinical microbiology laboratories in Ethiopia for routine antibiotic susceptibility testing and extended surveillance of multidrug resistance.

## Background

Bacterial production of extended-spectrum β-lactamases (ESBL) significantly reduces the efficacy of the most commonly used beta-lactam antibiotics for the empiric therapy of infections caused by putative Gram-negative bacteria [1]. While ESBL enzymes readily hydrolyse penicillins and cephalosporins, they have a far lower affinity for cephamycins and clavulanic acid [1, 2]. These hydrolytic enzymes are encoded by various gene variants. The major groups are TEM (Temoniera), CTX-M (Cefotaximase-Munich), SHV (Sulfhydryl variable), and OXA (oxacillin), all of which have been used for molecular detection of ESBL genes [3–5]. These genes are frequently mobile and located on plasmids and are thus transmitted horizontally [5]. These plasmids often contain mobile elements with resistance genes for additional drug classes such as sulfonamides, aminoglycosides and fluoroquinolones. Thus, bacteria carrying these plasmids are very often multi-drug resistant [6–8].

Gram-negative bacteria with the capacity to produce ESBL have become a serious global health problem, especially in resource-limited settings [9]. The rapid increase of ESBL-producing bacteria is associated with high mortality due to ineffective antibiotic treatment [10]. The management of patients with multidrug-resistant (MDR) bacteria requires well-staffed clinical units, reliable microbiology service and regular interaction between professional groups.

Due to limited financial resources and restricted supply chains, carbapenems are often unavailable in healthcare facilities in developing countries. Thus, the effective treatment of infections caused by ESBL-producing bacteria is limited, contributing to a high mortality [11].

In East Africa considerable variance in the prevalence of ESBL-producing Gram-negative bacteria between 13.4 and 89.0% has been described [12–14]. Understanding the epidemiology of ESBL at a country level is elemental to reinforce effective prevention and control strategies, but systematic surveillance of MDR pathogens in Ethiopia is non-existent. To assess the magnitude of the problem in Ethiopia, data on ESBL-producing bacteria in different regions of Ethiopia were extracted from existing publications, analyzed and summarized in this systematic review.

## Methods

To ensure inclusion of relevant information, the study was conducted based on the guideline of the Preferred Reporting Items for Systematic Reviews and Meta Analyses group checklist [15]. The outcomes of interest were the proportion of ESBL-producing bacteria among Gram-negative isolates from samples obtained from human patients in Ethiopia and the associated mortality.

### Study area

The study was conducted in Ethiopia; a country situated in the horn of Africa, covering a land area of 1.04 million km^2^. With a population of 110,14 million people, Ethiopia is the second most populous nation in Africa following Nigeria [16].

### Literature search and eligibility criteria

A systematic literature search was conducted on PubMed, PubMed Central, Medline, Science Direct and Google scholar, which are commonly used medical and biomedical databases in Ethiopia and accessible free of charge through different Ethiopian universities, to identify publications between January 1990 and March 2019 relevant for this review. No chargeable databases or databases without a medical focus were used for the literature search. The search strategy included all articles containing the descriptors. Structured search strategies were developed using the vocabulary terms of each database and targeting the “title” and “abstract” fields. The search was conducted by combining the following medical subject heading terms: “ESBL producing Enterobacteriaceae infections”, “Gram-negative infection associated mortality”, and “Ethiopia”, including all study types and populations. Additional publications were identified in the references of the initially identified articles, including systematic reviews and/or meta-analyses. Citation lists of publications meeting eligibility criteria for this meta-analysis were also reviewed.

In order to identify appropriate publications, the following selection criteria were used: a study had to (i) describe at least one pathogenic bacteria genus among the Gram-negative bacteria, (iii) be conducted on human subjects, (iii) isolate and identify bacteria from clinical specimens, (iv) test isolates for sensitivity to at least amoxicillin plus clavulanic acid and 3rd generation cephalosporins, (v) be conducted in Ethiopia, and (vi) be published in English. Therefore, reviews and publications not focusing on ESBL-producing Gram-negative bacteria isolated from human samples in Ethiopia and/or not subjected to antimicrobial sensitivity testing [17] were excluded. The titles and abstracts of the search results were reviewed and all publications were included that described cross-sectional, prospective, observational, and/or randomized controlled trials.

**Fig. 1.**
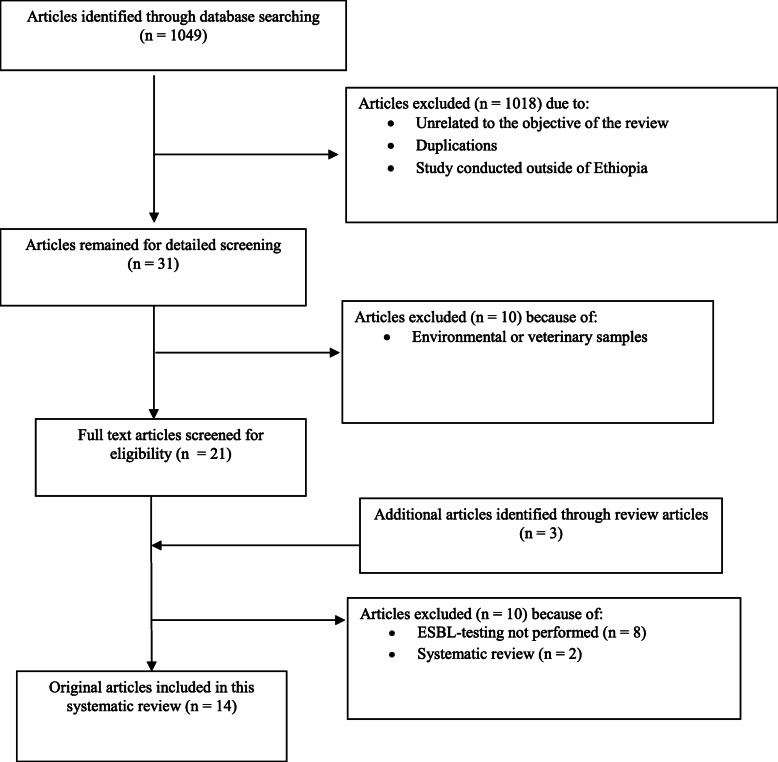
Flow diagram of the selection process for eligible studies.

The selection of publications followed three steps. First, titles of articles identified by literature search were checked for relevance of the studied topic. Publications, in which testing for ESBL was not performed, did not describe results from Ethiopia or not from clinical human samples and duplicate publications were discarded. Second, abstracts from selected publications were evaluated for the inclusion criteria. Third, the content of the remaining publications was accessed and evaluated for this review. Details of how eligible studies were included in the data synthesis is indicated in the flow diagram of the study selection process (Fig. 1).

### Description of studies

#### Types of studies

All randomized controlled clinical trials and cross-sectional studies investigating the drug susceptibility/resistance pattern of Gram-negative bacteria from Ethiopia published within the given time frame were included in the review.

#### Antimicrobial susceptibility testing

Within included studies the double disc synergy test (DDST) was used for phenotype detection of ESBL production showing a typical increase in growth inhibition by ceftazidime and cefotaxime in the zone adjacent to amoxicillin + clavulanic acid [18].

#### Study populations

The participants of included studies were distributed among all age, sex, and Ethiopian ethnic groups. Overall, 1049 published articles were reviewed. Of these, 14 original articles were included.

#### Outcome measurements

The main outcome measure was the proportion of ESBL-producing bacteria among Gram-negative isolates evaluated by drug susceptibility testing. Based on this, the isolates defined as resistant to the selected drugs were documented. Patient mortality associated with infections with ESBL-producing Gram-negative bacteria was also recorded.

### Data extraction and management

Data was collected from included studies with focus on the following characteristics: type of study, study population, antimicrobial sensitivity testing, and characteristic of the laboratory investigation. For data consistency, two researchers extracted data independently. Whenever there was discordance in the data extracted, consensus was reached by double-checking the article. From eligible studies, if available the following data were extracted: first author, year of publication, study period, study site, study design, population size, sample size, type of sample collection, methods used to test for ESBL, antimicrobial substances used for susceptibility testing, gene encoding for ESBL, number of Gram-negative bacteria isolated and tested for ESBL, the proportion of ESBL-producing bacteria among Gram-negative isolates, and mortality associated with ESBL-producing bacterial infection.

### Data synthesis and analysis

The mean proportion of ESBL-producing bacteria was calculated, using the sum of the numbers of ESBL-producing Gram-negative bacteria in all studies considered, divided by the sum of the number of Gram-negative bacteria tested for ESBL. The pooled proportion estimates for ESBL-producing Gram-negative bacteria in the general population and their 95% CI were calculated using the random effects model meta-analysis [19].

Heterogeneity between studies was evaluated through the Cochran’s Q test (reported as *p* value) and inverse variance index (I^2^) [20].

For each study, the prevalence with corresponding 95% CI and the overall random effects pooled estimate of all the studies were presented.

Data was analyzed using RStudio Version 1.1.456 –© 2009–2018 (RStudio Inc., Boston, MA, USA). A map showing the number of studies and prevalence of ESBL- producing Gram-negative bacteria in different region of Ethiopia was created, using Quantum GIS software version 2.0.1 (Open Source Geospatial Foundation, Boston, USA).

## Results

### Distribution of articles describing ESBL in Ethiopia

The initial database search returned 1049 abstracts. Of those, 1018 publications were discarded after reviewing their titles. A further 10 articles either described environmental or veterinary samples or ESBL-producing bacteria were not identified and described in the articles. The full text of 21 articles and an additional three articles identified through review articles were scrutinized for eligibility. Of these, 8 were excluded because ESBL phenotypes or genotypes were not clearly described and 2 publications were identified as systematic reviews. Finally, 14 articles fulfilled eligibility criteria and were subjected to meta-analysis [8, 21–33] (Fig. 1).

**Fig. 2:**
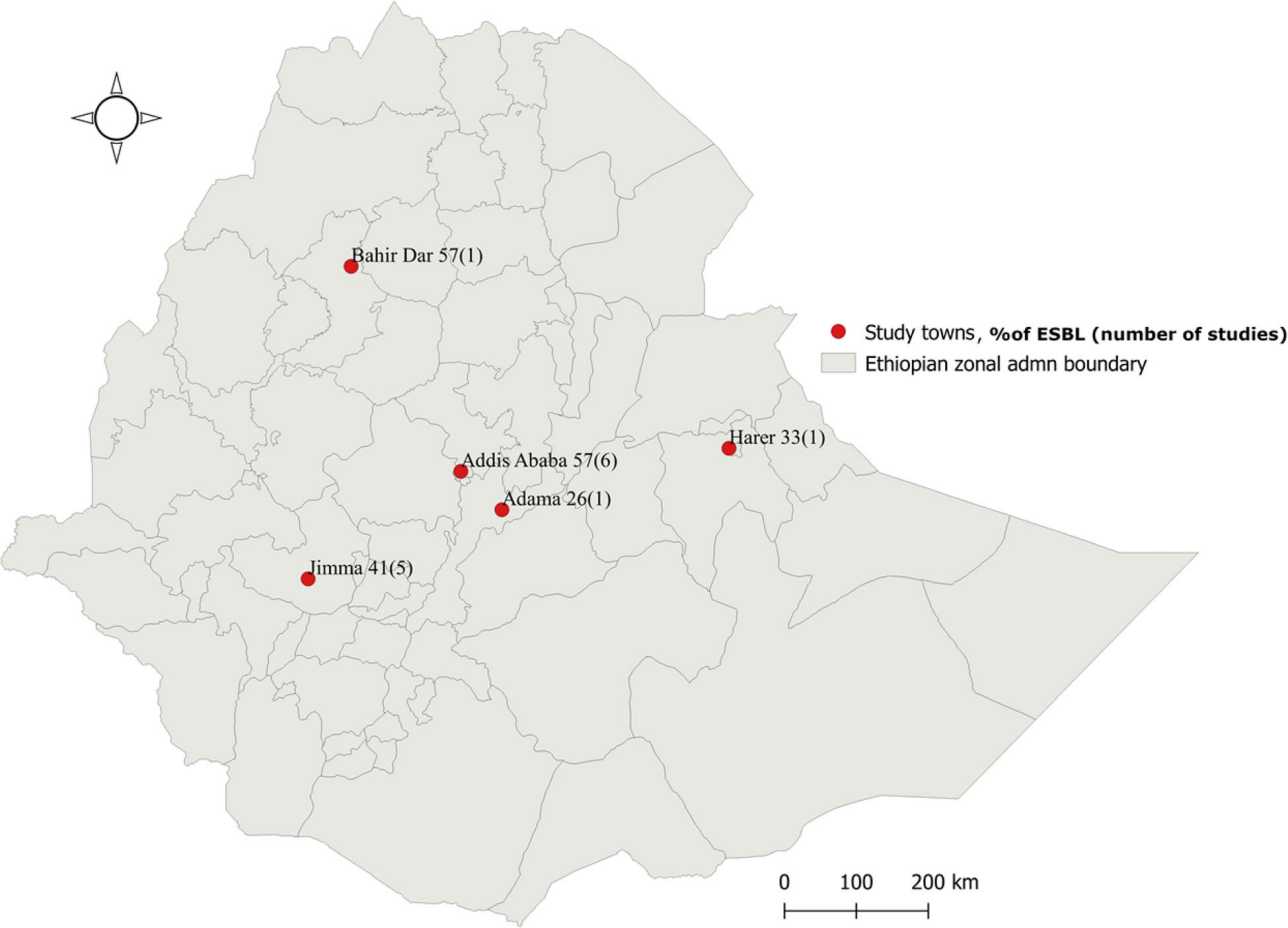
Prevalence and number of eligible articles published on ESBL-producing Gram-negative bacteria in different regions of Ethiopia, March 2019

In total, the 14 articles reviewed described cross-sectional hospital-based studies. Of those, 6 (43.0%) were published from the cities of Addis Ababa, 5 (36.0%) from Jimma, and 1 (7%) from each of the following: Adama, Bahir Dar, and Harar (Fig. 2). All reviewed articles included patients attending in-patient and/or outpatient departments. A total of 5191 samples from human study participants were analyzed including urine (*n* = 1273), stool (*n* = 1679), swabs (*n* = 266), sputum (*n* = 294), blood cultures (*n* = 192) and body fluids (*n* = 34) (Table 1).

**Table 1:** Summary of the 14 studies reporting the prevalence of ESBL-producing Gram-negative bacteria in different parts of Ethiopia, 2005–2019

Author, year	Study period	Study area (site)	Sample size	Sample types (Culture specimens)	No. of Gram-negative isolates	No. of ESBL (%)
Abayneh M et al., 2018 [32]	March to June 2016	Jimma	342	urine	74	17 (23.0)
Abera B et al., 2016 [33]	Sept 2013 to March 2015	Bahir Dar	477	blood, urine, pus/swab and body fluids	210	120 (57.1)
Beyene G et al., 2011 [21]	Jan to Aug. 2006	Addis Ababa	1225	stool, blood	113	78 (69.0)
Desta K et al., 2016 [22]	10–20 Dec 2012	Addis Ababa	267	stool	295	151 (51.2)
Eugale T et al., 2018 [23]	April 2013 to March 2014	Addis Ababa	68	Stool	68	12 (17.6)
Gashaw M et al., 2018 [24]	May to Sept. 2016	Jimma	197	urine, swab/pus, blood, and sputum	100	36 (36.0)
Legese et al., 2017 [25]	Jan. to March 2014	Addis Ababa	322	blood and urine	28	22 (78.6)
Mulisa et al., 2015 [26]	April to Aug. 2016	Adama	384	urine, stool, swabs, and body fluids	65	17 (26.0)
Mulualem Y et al., 2012 [27]	Feb. to March 2007	Jimma	359	urine, stool, swabs, and sputum	67	24 (35.8)
Pritsch M et al., 2017 [28]	Jan. 2014 to June 2015	Jimma	224	swabs and body fluids	14	3 (21.4)
Seboxa T et al., 2015 [29]	Aug. 2012 and Oct. 2013.	Addis Ababa	292	blood	20	9 (45.0)
Seid & Asrat, 2005 [30]	Dec. 2003 to Feb. 2004	Harar	384	sputum, urine and pus	57	19 (33.3)
Teklu DS et al., 2019 [31]	Jan. to May 2017	Addis Ababa	426	urine, blood, swabs, and body fluids	426	246 (57.7)
Zeynudin A et al., 2018 [8]	March to Oct. 2014	Jimma	224	urine, swabs, blood and fluids	112	71 (63.4)

### Laboratory methods used to detect ESBL-producing bacteria

Eleven (78.6%) of the reviewed articles used the double disk synergy test (DDST) alone, while the remaining 3 (21.4%) articles used both DDST and polymerase chain reaction (PCR)-based molecular methods to investigate the proportion of ESBL-producing bacteria.

Selected articles were published from 2005 to 2019. Inclusion of study participants ranged from 2003 to 2017. All data in the included publications were obtained from tertiary hospitals. The highest proportions of ESBL-producing bacteria were reported from the capital city, Addis Ababa (57%) and Bahir Dar, the capital of Amhara Region in the northwestern part of Ethiopia (57%). The lowest proportion was reported from Adama in Oromia Region / central Ethiopia (25%) (Fig. 2).

**Fig. 3:**
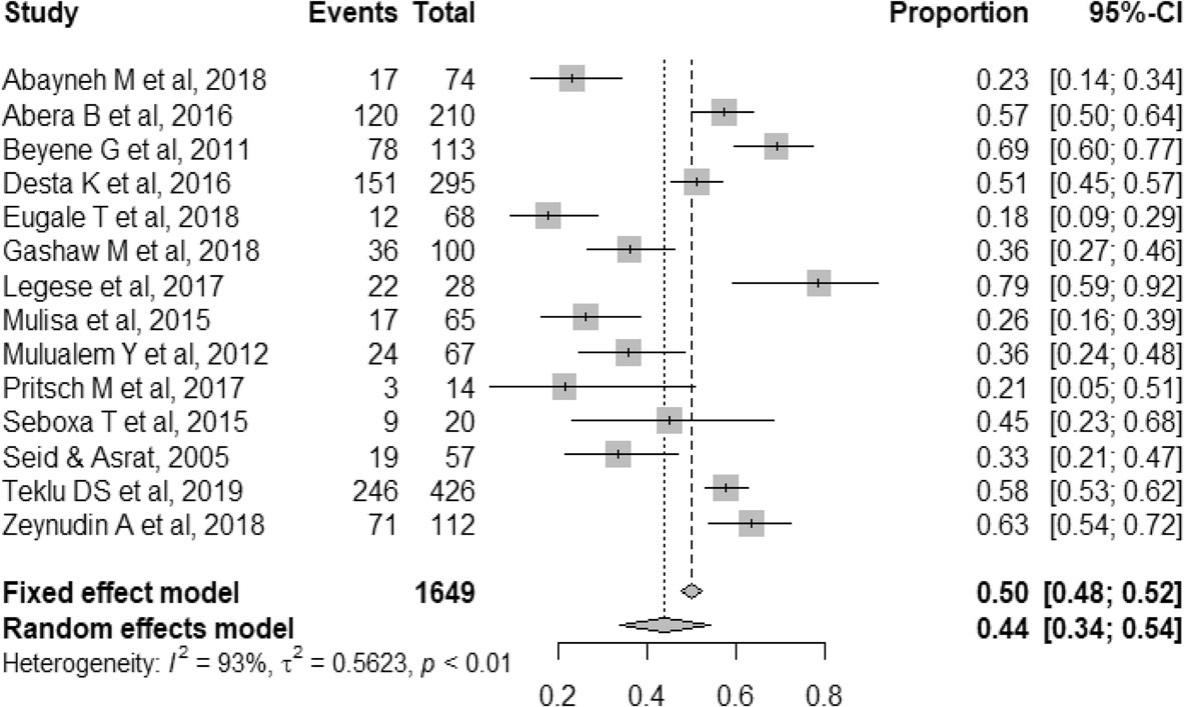
Forest plot of pooled prevalence of ESBL-producing Gram-negative bacteria in 14 studies, Ethiopia, 2005–2019

As presented in the forest plot (Fig. 3) the pooled proportion of ESBL-producing Gram-negative bacterial isolates from human samples in Ethiopia was 50% (95% CI: 0.48–0.52). Random model methods showed a high level of heterogeneity (I^2^ = 95%, *p* <  0.01). Concerning regional differences, the pooled proportion of ESBL-producing Gram-negative bacteria in Addis Ababa was 56.5% (95% CI, 0.53–0.60; I^2^ = 95.2% and *p* <  0.0001) compared to 41% (95% CI, 0.36–0.46; I^2^ = 84.6% and p <  0.0001) in the city of Jimma in the southwestern part of Ethiopia.

The studies included were conducted in different clinical settings, study periods, with differing clinical samples and study populations. This might be an influencing factor for the heterogeneity of the results.

In total, 1649 Gram-negative bacteria isolated from 5191 clinical samples were included. The pooled rate of ESBL-producing Gram-negatives was determined to be 50.1% (95% CI: 47.7 –52.5%). Among different species, ESBL production rates were 65.7% (*n* = 263) for *Klebsiella* spp., 62.2% (*n* = 33) for *Enterobacter* spp., 48.4% (*n* = 90) for *Salmonella* spp., 47.0% (*n* = 383) for *E. coli*, 46.8% (*n* = 22) for *Citrobacter* spp., 43.8% (*n* = 7) for *Providencia* spp*.,* 28.3% (*n* = 15) for *Proteus* spp., 17.4% (*n* = 4) for *Pseudomonas aeruginosa*, 9.4% (n = 3) for *Acinetobacter* spp*.,* and 20.8% (*n* = 5) for other Gram-negative bacteria, respectively (Table 2).

**Table 2:** Species distribution of the pooled proportion of ESBL-producing Gram-negative bacteria

Gram-negative bacteria	No. of studies	No. of isolates	No. of ESBL-positive isolates	ESBL Proportion [95% CI]	I^2^ (*p*-value)
*Klebsiella* spp*.*	10	400	263	0.66 [.61; .70]	< 0.001
*Enterobacter* spp*.*	6	53	33	0.61 [.47; .74]	< 0.001
*Salmonella* spp*.*	5	186	90	0.48 [.33; .63]	< 0.001
*E. coli*	10	815	383	0.47 [.44; .50]	< 0.001
*Citrobacter* spp*.*	5	47	22	0.47[.33;,63]	0.001
*Providencia* spp*.*	3	16	7	0.44 [.20; .70]	0.011
*Proteus* spp*.*	6	53	15	0.28 [.16; .42]	0.053
*Pseudomonas aeruginosa*	2	23	4	0.17 [.05; .39]	0.386
*Acinetobacter* spp*.*	3	32	3	0.09	–
Others^a^	5	24	5	0.09 [.02;.25]	0.142
Total		1649	825	0.25 [.09; .49]	< 0.0001

^a^Others: *Alcaligenes faecalis* (1 ESBL of 3 isolates), *Morganella morganii* (3 ESBL of 12 isolates); *Stenotrophomonas maltophilia (*1 ESBL of 4 isolates), *Escherichia hermanii* (1 ESBL of 1 isolates)

### Molecular epidemiology of ESBL in Ethiopia

Out of 14 reviewed articles, only three (21.4%) studies, one from Addis Ababa University Black Lion Hospital and two from Jimma University Specialized Hospital, reported on the underlying resistance genes for the detection of ESBL resistance [8, 23, 28]. A total of 81 isolates were molecular-biologically analyzed for genetic resistance mechanism. All of those were positive for ESBL-encoding genes: 82.7% (67/81) carried CTX-M-1 group and 17.3% (14/81) TEM. All CTX-M-1 group ESBL genes reported from included studies were confirmed to be CTX-M-15 genes [8, 23]. Additional genes detected were: one isolate carrying a *bla*_OXA_ gene from Addis Ababa, two *bla*_CTX_ of the CTX-M-9 group [23] and three *bla*_SHV_ genes only expressed in *Enterobacter cloacae* isolated in Jimma [8].

### Outcome of patients infected with ESBL-producing bacteria

Only two of the analyzed studies reported the mortality rate associated with infections by ESBL-producing pathogens or resistance to 3rd generation cephalosporins. The pooled mortality within these publications was 86% (12/14) [28, 29]. One study from Black Lion Teaching Hospital in Addis Ababa reported a high mortality among patients with Gram-negative bacterial blood stream infection, particularly among patients infected with multi drug resistant bacteria. Twelve out of 20 patients with Gram-negative sepsis died in the hospital. Of those, 91.7% (11/12) were infected with ESBL-producing bacteria [29].

## Discussion

Infections caused by ESBL-producing Gram-negative bacteria are increasing at an alarming rate and have become a serious public health threat worldwide. Summarized data of the burden of ESBL-associated antibiotic resistance is limited in Africa. To our knowledge, this is the first systematic review of data from Ethiopia concerning ESBL-producing bacteria from clinical specimens.

In this study, the pooled prevalence of ESBL-producing Gram-negative bacteria was 50.1%. This prevalence lies in the middle of a wide range between 13.4 to 89.0% reported in other studies from East Africa [13, 14]. Among the different species identified and investigated in the studies, *Klebsiella* spp. were the most frequent ESBL-producing Gram-negative bacteria followed by *Enterobacter* spp., *Salmonella* spp., *E. coli* and *Citrobacter* spp. (Table 2). Similar findings have been reported from Uganda, with ESBL-rates of 52% for *Klebsiella spp.* and 44% for *E. coli* [12]. One recent study from northern Ethiopia also confirmed the highest rate of ESBL-production among Gram-negative isolates from human samples in *K. pneumoniae* [34].

The most common ESBL-producing non-fermenting Gram-negative rods (NFGN) are *Pseudomonas aeruginosa* and *Acinetobacter* spp. [35]. In this review, we identified a relatively low ESBL rate among NFGN bacteria compared with the *Enterobacteriales* of 17.4 and 9.4% for *Pseudomonas aeruginosa* and *Acinetobacter* spp*.,* respectively (Table 2). One study from Jimma, in southwest Ethiopia described a high prevalence of *Acinetobacter* spp., during screening for possible ESBL-production using 3rd generation cephalosporins alone. However, genotyping results confirmed a low proportion of ESBL production in *Acinetobacter* spp*.* at the study site [8]. This shows that genotypic screening for MDR in NFGN might not be beneficial. In this case, the functional and more general approach of phenotypic resistance testing seems superior.

Molecular ESBL gene detection is not commonly practiced in Ethiopia. From all studies included in this review, gene detection was performed for 81 strains out of the 825 ESBL-producing Gram-negative bacteria determined by DDST. The CTX-M-1 group ESBL genes were most frequently detected, with all of these identified as CTX-M-15, followed by *bla*_TEM_. Reports from India showed similar results, in which the prevalence of *bla*_CTX-M_ was highest (82.5%), followed by *bla*_TEM_ (67.5%) and *bla*_SHV_ (57.5%) among the ESBL-genes identified from clinical Gram-negative isolates [17]. In the studies included in this review, *bla*_SHV_ was only detected in three *Enterobacter cloacae* isolates*.* Only a single isolate carrying a *bla*_OXA_ gene from Addis Ababa [24] and two isolates expressing CTX-M-9 group genes from Jimma [8] were described. More studies are needed to establish a comprehensive overview of the distribution of ESBL genes in Ethiopia.

Isolates of *Acinetobacter baumannii* carrying the carbapenem resistance gene NDM-1 (New Delhi metallo-beta-lactamase) were reported from Jimma [﻿35﻿]. In East Africa, the NDM-1 gene was first detected from isolated *Acinetobacter baumannii* in Kenya [36]. However, the current data on the prevalence of carbapenem resistance in East Africa ranges from 1% in the Democratic Republic of Congo to 35% in Tanzania [37], although no systematic surveillance is conducted in these countries and thus, as in Ethiopia, the true prevalence is unknown.

Pooled data currently available suggest that infections caused by ESBL-producing bacteria are leading to prolonged hospital stays and are associated with an increased risk of mortality [11, 38]. Even though available data from Ethiopia is very limited concerning the outcome of patients infected with ESBL-producing bacteria, one study from Addis Ababa describes a mortality rate of 100% in 11 patients [29]. In patients with sepsis caused by Gram-negative bacteria, mortality is strongly associated with antibiotic resistance [39, 40]. The absence of timely microbiology reports in patients with Gram-negative bacterial sepsis compromises successful antimicrobial treatment and possibilities for antimicrobial stewardship. However, even if pertinent resistance patterns would be available earlier, the frequent unavailability and high prices of effective antibiotics greatly contributes to morbidity and mortality associated with these infections.

As a study conducted in Tanzania indicates, inappropriate antibiotic use and two-week fatality rates were significantly higher among patients with septicemia due to ESBL-producing organisms than among those with infections due to non-ESBL producing bacteria [40]. The 30-day and 90-day survival rates of patients infected by MDR bacteria were also significantly lower than patients infected by non-MDR bacteria (58.8% vs. 75.0% at 30 days and 43% vs. 63%, at 90 days) [41]. In a study published in the UK, mortality due to blood stream infections caused by ESBL-producing *E. coli* was significantly higher than from non-ESBL *E. coli* [42]*.* In countries and regions with high rates of ESBL carrying bacteria, as demonstrated here for Ethiopia, these findings have serious implications for empirical antibiotic prescription practice. In Ethiopia, cephalosporins should be considered ineffective in up to 66% of infections with *Klebsiella* spp. and 47% of cases of *E. coli* infections as was reported in 2007 for ESBL-*E. coli* [42].

In order to guide empiric therapy, continuous surveillance of common resistance patterns of Gram-negative isolates and monitoring of ESBL genes circulating in the population are essential. For this task, clinical microbiology laboratories need to be established, upgraded and maintained. In this review, CTX-M-15 ESBL genes were the most frequently detected ESBL gene. At this time, this gene could be used as target for ESBL screening programs in Enterobacteriales in the country, where high-quality and reliable bacteriological laboratories are still rather rare. In general, the molecular testing for *bla*_CTX-M-1_ or *bla*_CTX-M-15_ would provide a reliable prediction of overall resistance due to ESBL [43]. In order to reduce the dissemination of antibiotic resistance within the country, infection prevention and control measures and the establishment of antimicrobial stewardship programs should be strengthened.

This review is limited by the fact that available literature does not permit a meta-analysis of adjusted mortality associated with infections caused by ESBL-producing bacteria, as only two of the 14 included studies reported the results of patient outcome. Thus, only crude mortality but neither attributable mortality, nor causality were described. Therefore, this meta-analysis highlights the deficiencies of the existing literature to calculate adverse outcome attributable to ESBL-associated resistance. Because of possible clinical implications it would be interesting to describe the proportion of ESBL-positive Enterobacterales in the different clinical specimen (blood, urine. Etc.). However, it was not possible to list proportions of ESBL-producing isolates in different clinical specimen, because it was not stated respective of clinical samples in most articles included into this study. Since ESBL genotypes were only reported in two studies, the pooled prevalence of infections due to ESBL phenotype in Ethiopia was analyzed.. Clinical Laboratory Standards Institute (CLSI) or European Committee on Antimicrobial Susceptibility Testing (EUCAST) guidelines were used to interpret AST results by all investigators of studies included in this review.

## Conclusion and recommendations

In this meta-analysis, the pooled prevalence of phenotypic ESBL production among Gram-negative isolates from human samples is remarkably high. Despite the scarcity of data, infections caused by ESBL-producing bacteria are very likely resulting in an increased mortality. In resource-limited settings, double-disk synergy test can be implemented for screening of ESBL production. The CTX-M-1 group is the predominantly detected ESBL genotype with all of the detected genes confirmed to be CTX-M-15 genes. The CTX-M-1 group or CTX-M-15 gene could therefore be targeted for rapid genetic ESBL screening in the country, thus providing early essential information for decisions on appropriate and effective treatment. In order to provide physicians with urgently needed guidance for antimicrobial therapies according to detected antimicrobial resistance patterns, establishment, upgrading and maintaining of clinical microbiology laboratories in the country capable of reliable double-disk synergy testing are essential. The possibility of ESBL gene detection for routine surveillance is desirable. National and regional treatment guidelines should be based upon MDR surveillance to effectively treat patients and prevent the spread of resistance genes in hospitals and communities. Emphasis for production of antibiotic substances in the country or import from abroad should consider recent MDR surveillance data.

## Data Availability

All data generated or analysed during this study are included in this published article.

## Abbreviations

AST: Antimicrobial susceptibility testing
CI: Confidence interval
CLSI: Clinical Laboratory Standards Institute
CTX-M: Cefotaximase-Munich
DDST: Double disc synergy test
ESBL: Extended spectrum beta-lactamase
EUCAST: European Committee on Antimicrobial Susceptibility Testing
MDR: Multidrug-resistant
NDM: New Delhi metallo-beta-lactamase
NFGN: non-fermenting Gram-negative rods
OXA: Oxacillin
PCR: polymerase chain reaction
SHV: Sulfhdryl variabl
TEM: Temoniera
