# Correction to: High rate of extended-spectrum beta-lactamase-producing gram-negative infections and associated mortality in Ethiopia: a systematic review and meta-analysis

**DOI:** 10.1186/s13756-020-00806-6

**Published:** 2020-08-22

**Authors:** Tafese B. Tufa, Andre Fuchs, Takele B. Tufa, Loraine Stötter, Achim J. Kaasch, Torsten Feldt, Dieter Häussinger, Colin R. Mackenzie

**Affiliations:** 1 Hirsch Institute of Tropical Medicine, P.O. Box 04, Asella, Ethiopia; 2 College of Health Sciences, Arsi University, P.O. Box 04, Asella, Ethiopia; 3 Department of Gastroenterology, Hepatology and Infectious Diseases, Duesseldorf University Hospital Center, Moorenstr. 5, 40225 Duesseldorf, Germany; 4 College of Veterinary Medicine and Agriculture, Addis Ababa University, Bishoftu, Ethiopia; 5 Institute of Medical Microbiology and Hospital Hygiene, Düsseldorf University Hospital Centre, Universitätsstr. 1, 40225 Düsseldorf, Germany; 6 Institute of Medical Microbiology and Hospital Hygiene, Magdeburg University Hospital, Otto-von-Guericke-University, Leipziger Str. 44, 39120 Magdeburg, Germany

**Correction to: Antimicrob Resist Infect Control 9, 128 (2020)**

**https://doi.org/10.1186/s13756-020-00782-x**

After publication of this article [1], it is reported that one of the author names is incorrect:

‘Torsten Feld’ should be corrected to ‘Torsten Feldt’. The author name has thus been updated in this Correction.

The original article has also been updated.
